# Cystoscopic Findings in a Case of Purpura Hemorrhagica

**Published:** 1914-01

**Authors:** Ferdinand C. Walsh

**Affiliations:** San Antonio, Texas


### Cystoscopic Findings in a Case of Purpura Hemorrhagica.

BY FERDINAND C. WALSH, M. D., SAN ANTONIO, TEXAS.

Purpura hemorrhagica is of sufficiently rare occurrence to warrant reporting the following case, particularly as the cutaneous symptoms on which a positive diagnosis is usually based did not become manifest until late in the course of the disease.

January 24, 1913, in consultation with Dr. Thos. Dorbandt, I first saw the patient, Miss A. J., school teacher, aged 23 years. She was the second of five children, the rest of whom are living and in good health. Previous history, except for the diseases of childhood, negative.

During November, 1912, an intermittent hematuria developed, the urine for several days at a time showing the presence of blood. Accompanying the hematuria there was a mild grade of cystitis which disappeared as the urine became free from blood. After the hematuria had been present off and on for several weeks, during which time the patient rapidly lost weight and strength, nausea and frontal headache developed; dyspnea was occasionally present, and anemia and nervousness became very marked. An anemic heart murmur was apparent, and edema of the lower extremities appeared. December 12th, urinalysis was negative save for the presence of a small amount of sugar. December 18th, the urine showed absence of sugar but presence of a large amount of blood. December 22d, urinalysis was negative.

From December 22d, until my first observation of the case on January 27, 1913, the patient rapidly lost ground, the hematuria being almost constantly present. On this date, physical examination showed marked anemia with emaciation. Abdominal palpation was negative except for a tender area in the right hypochondrium. The tenderness was most pronounced when pressure was exerted bimanually, one hand being placed in the dorso-lumbar region, the other just below the hepatic border in front. The right kidney in this manner could be palpated, and seemed to be the seat of tenderness. There was an appreciable anlargement, and from the absence of symptoms on the opposite side, it was presumed that the hematuria arose from a disturbance of some kind, possibly a hypernephroma, in the right kidney. Preparation for a cystoscopic examination, in order to determine the source of the bleeding and the relative condition of both kidneys, was made, but the patient refused to submit to further examination at the time.

On February 18th, her condition having become much graver, the patient consented to a cystoscopy, to be followed, if necessary, by operation. Under ether anesthesia, the cystoscope was easily introduced. Bladder capacity, normal. The mucosa throughout, but particularly that covering the fundus and lateral walls, was studded with punctate hemorrhagic areas, none of which at the time was actively bleeding but which showed that bleeding had recently taken place therefrom. The urine for several hours previous to the examination had not contained blood microscopically. Catheters were placed in the ureters, urine from both kidneys being obtained. The urine from both sides appeared normal and it was evident that the source of blood previously found was from these hemorrhagic spots in the bladder mucosa. February 2d, the patient developed typical purpuric hemorrhages of the skin, the areas varying in size from that of a pea to an inch in diameter. In spite of such treatment as is usually directed to this condition, embracing calcium chloride, sulphurous acid, gelatine, fruit acids, horse serum, and individual blood serum by transfusion, the patient died from exhaustion, February 18th.—*Journal of Surgery,* September, 1913.

